# Bacteremia caused by *Helicobacter trogontum* indicative of zoonotic infection in a pig farmer: a case report

**DOI:** 10.1128/asmcr.00002-25

**Published:** 2025-07-09

**Authors:** Nobumasa Hojo, Takashi Unehara, Masato Suzuki, Michio Suzuki, Emiko Rimbara

**Affiliations:** 1 Department of General Medicine, NHO Hamada Medical Center 26817 , Hamada City, Shimane, Japan; 2 Department of Clinical Laboratory, NHO Okayama Medical Center, Okayama City, Japan; 3 Antimicrobial Resistance Research Center, National Institute of Infectious Diseases 13511, Tokyo, Japan; 4 Department of Veterinary Science, National Institute of Infectious Diseases 13511, Tokyo, Japan; 5 Department of Bacteriology II, National Institute of Infectious Diseases 13511, Tokyo, Japan; Pattern Bioscience, Austin, Texas, USA

**Keywords:** *Helicobacter trogontum*, bacteremia, enterohepatic *Helicobacter *species, non-*pylori*, zoonosis

## Abstract

**Background:**

*Helicobacter pylori* was first isolated from humans in 1983. Since then, >50 *Helicobacter* species have been registered. *Helicobacter trogontum* was first isolated from rat colonic mucosa in 1996 and has been isolated from pig feces and the livers of aborted sheep. *H. trogontum* adheres to and invades human cells and secretes factors that may contribute to disease development.

**Case Summary:**

A 41-year-old woman, who worked on a pig farm, presented to our hospital with sudden-onset headache, nausea, general fatigue, chills, and fever. Plain computed tomography revealed small lymph nodes in the ileocecal region. Intravenous ceftriaxone, vancomycin, and acyclovir were administered for suspected meningoencephalitis. She was discharged from the hospital on day 9 after her symptoms improved. The cerebrospinal fluid culture was negative. However, an aerobic bottle was positive in one of two blood culture sets. Microscopic examination with Gram staining revealed fusiform gram-negative bacteria. Whole genome sequencing of the NHP16-4001 isolate confirmed *H. trogontum* infection.

**Conclusion:**

Case reports of human infection with *H. trogontum* are rare. However, *H. trogontum* has been reported to cause enterocolitis and sepsis in an immunocompetent human, and skin lesions as well as bacteremia in an immunocompromised human. *H. trogontum* was isolated from pigs, sheep, and mice, suggesting its potential importance as a zoonotic disease. In this case, we hypothesize that bacteremia development was related to contact with pig feces on the patient’s farm. Thus, *H. trogontum* infection may be considered a zoonosis; however, further reports are warranted for arriving at a definitive conclusion.

## INTRODUCTION

Since the first isolation of *Helicobacter pylori* from humans in 1983, >50 *Helicobacter* species have been registered according to the Taxonomy of the National Center for Biotechnology Information (NCBI). *Helicobacter* species in the stomach, such as *H. pylori* and *H. felis*, are classified as gastric *Helicobacter* species, while those in the biliary or intestinal tract, such as *H. cinaedi* and *H. hepaticus*, are enterohepatic *Helicobacter* species. Enterohepatic *Helicobacter* species have been reported to be associated with gastroenteritis, hepatitis, bacteremia, and other diseases in humans (1, 2). *H. trogontum* was first isolated from rat colonic mucosa in 1996 and, since then, has been isolated from pig feces and the livers of aborted sheep (3–5). *H. trogontum* can adhere to and invade human cells; it secretes factors that may contribute to disease development (6). Case reports of human infections with *H. trogontum* are rare. Despite this, *H. trogontum* infection appears to be a potentially important zoonotic disease as the bacteria can be found in rats, pigs, and sheep. Herein, we discuss a case of bacteremia caused by an enterohepatic *Helicobacter* species in an immunocompetent woman working on a pig farm.

## CASE PRESENTATION

A 41-year-old woman presented to our emergency department with sudden-onset headache, nausea, general fatigue, chills, and fever. She had no relevant medical history and was working on a pig farm with over 1,000 swine, where she directly cared for the sows and piglets, including feeding and fecal disposal.

**Fig 1:**
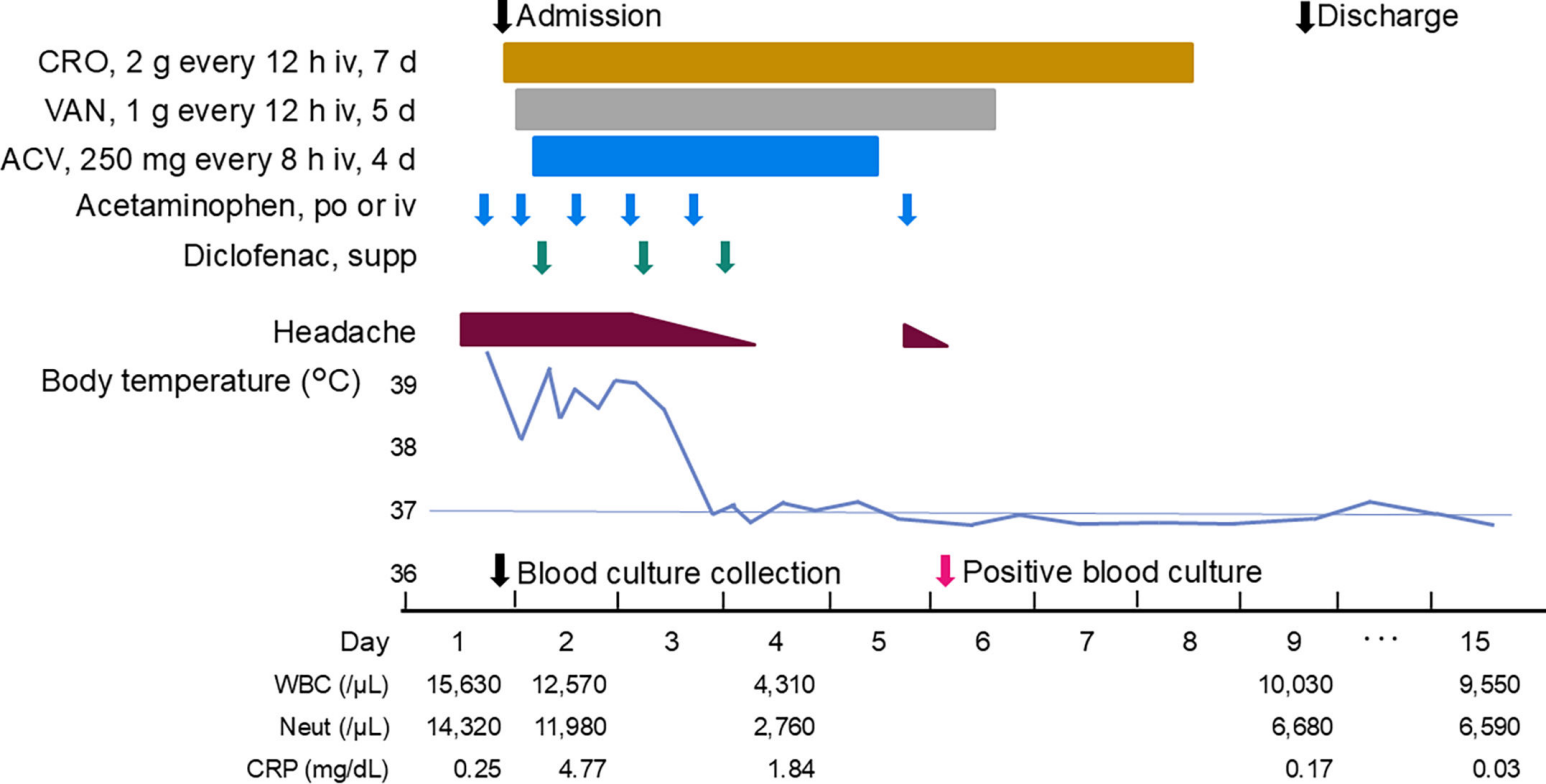
The patient’s clinical course. From the top, the chart shows the dates of admission and discharge, medication details, headache condition, changes in body temperature, dates of blood culture collection and positive results, and changes in white blood cell counts, neutrophil counts, and level of C-reactive protein. Abbreviations: ACV, acyclovir; CRO, ceftriaxone; CRP, C-reactive protein; iv, intravenous; Neut, neutrophil count; po, per os; supp, rectal suppository; VAN, vancomycin; WBC, white blood cell count.

On admission (Fig. 1), her body temperature was elevated at 40.1°C (reference range: 35.5–37.0), blood pressure was 103/49 mmHg (reference range: 100–140/60–90), pulse rate was 99 beats/min (reference range: 50–100), respiratory rate was 21 /min (reference range: 10–15), and oxygen saturation was 96% in ambient air (reference range: ≥96), with no obvious abnormalities. Her Glasgow Coma Scale was E4V4M6 (normal: E4V5M6); she was confused and disoriented but able to answer questions. Nuchal rigidity and Kernig’s sign were negative; jolt accentuation was positive; thus, the presence of meningitis could not be ruled out.

Two sets of aerobic and anaerobic blood cultures were collected 1 hour after presentation to the emergency department. Cerebrospinal fluid (CSF) examination revealed that the CSF appeared watery and colorless, with a low initial pressure of 24 mmH_2_O (reference range: 70–150), cell count of 1 /µL (reference range: ≤5), total protein of 32 mg/dL (reference range: 15–45), and glucose of 54 mg/dL (reference range: 45–80). The patient had a peripheral white blood cell count of 15,630 /µL (reference range: 3,300–8,600 /µL), 91.6% neutrophils (reference range: 40%–70%); C-reactive protein level of 0.25 mg/dL (reference range: 0.00–0.14 mg/dL); and procalcitonin level of 0.22 ng/mL (reference range: 0.00–0.49 ng/mL). Plain computed tomography revealed small lymph nodes in the ileocecal region. Therefore, we initially suspected meningoencephalitis or bacteremia and administered intravenous ceftriaxone (2 g every 12 hours), vancomycin (1 g every 12 hours), and acyclovir (250 mg every 8 hours) for 7, 5, and 4 days, respectively. On day 6, gram-negative bacteria were detected in one of the two blood bottles. Because it took a long time for the culture to become positive and only one set was detected, the result was initially determined to be contamination. She was discharged on day 9 after her symptoms improved (Fig. 1).

**Fig 2:**
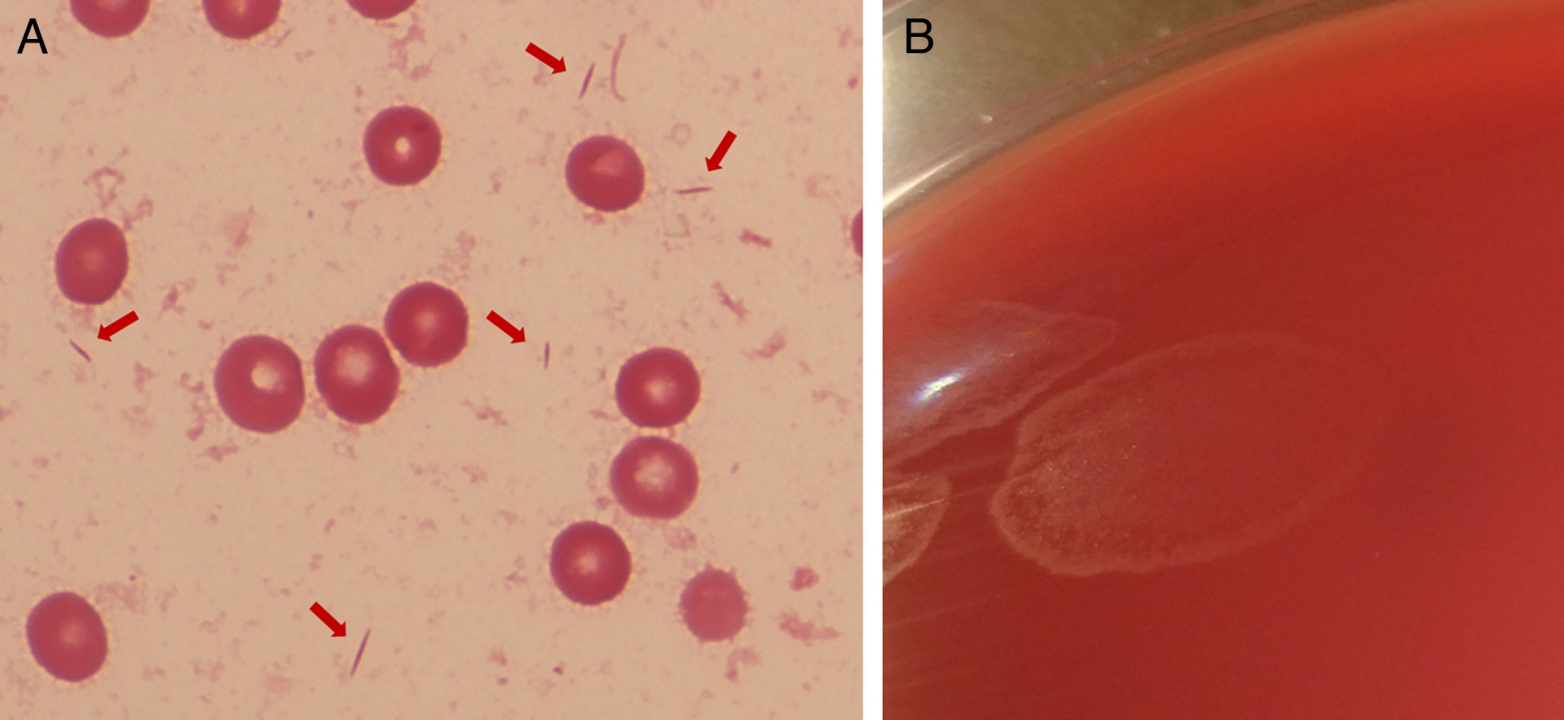
(**A**) Gram stain showing fusiform gram-negative bacilli from the positive blood culture bottle after 98 hour incubation (×1,000). (**B**) Swarming colonies on the blood agar medium after 4 days of subculture.

**Fig 3:**
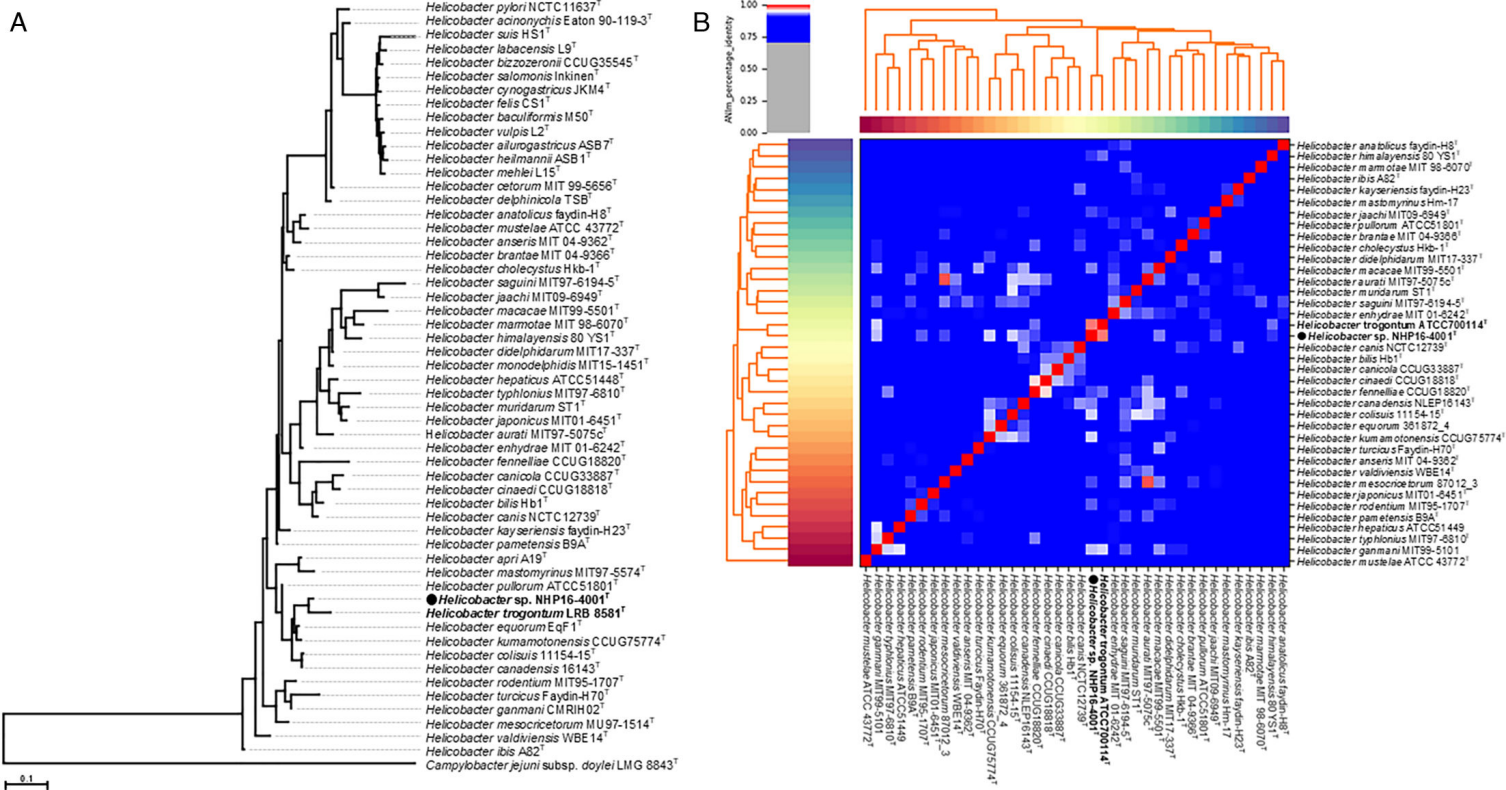
Phylogenetic trees and average nucleotide identity identifying *Helicobacter* sp. NHP16-4001 as *H. trogontum*. (**A**) Phylogenetic tree generated from the 16S rRNA gene of *Helicobacter* species and *Helicobacter* species strain NHP16-4001. Sequences were aligned using MAFFT version 7.49, and the phylogenetic tree was constructed using RAxML-NG version 1.1.0 with a GTR + G + I model and 100 bootstrap replicates. *Campylobacter jejuni* subsp. *doylei* was included as an out-group. The scale bar indicates the number of base substitutions per site. The 16S rRNA gene sequences of type strains of *Helicobacter* species were obtained from NCBI (https://www.ncbi.nlm.nih.gov/) via the links shown in List of Prokaryotic names with Standing in Nomenclature (LPSN, https://www.bacterio.net/). (**B**) Calculation of the average nucleotide identity among enterohepatic *Helicobacter* species and *Helicobacter* species strain NHP16-4001 using Pyani 0.2.12. A heatmap of hierarchical clustering analysis is shown. Pairs of genomes with ANI >95% are considered to be from the same species. The genomic sequences of the type strains of *Helicobacter* species were obtained from NCBI (https://www.ncbi.nlm.nih.gov/datasets/genome/).

The PCR test result for *Mycobacterium tuberculosis* and varicella-zoster virus antibody in the CSF was negative, and the CSF culture results, including that for *M. tuberculosis*, were negative. The bacteria detected from blood cultures were examined in more detail. Blood cultures were positive in only one aerobic bottle of two sets after 98 hours of incubation. Gram stain of the positive blood culture broth revealed fusiform gram-negative bacteria (Fig. 2A). The bacterial isolate produced swarming colonies after 4 days of subculture**,** which were oxidase and catalase positive (Fig. 2B). To further identify the isolate (named NHP16-4001), whole genome sequencing (WGS) was performed using the MiniSeq platform (Illumina, SD, USA). The reads were assembled using Shovill v1.1.0 (https://github.com/tseemann/shovill) and annotated using the DFAST server (https://dfast.ddbj.nig.ac.jp). The 16S rRNA gene of NHP16-4001 has 97% identity with six *Helicobacter* species: *H. equorum*, *H. kumamotonensis*, *H. trogontum*, *H. pullorum*, *H. canadensis*, and *H. colisuis*. Phylogenetic analysis of the 16S rRNA gene revealed that NHP16-4001 is most closely related to *H. trogontum* (Fig. 3A). The calculation of the average nucleotide identity among enterohepatic *Helicobacter* species using Pyani 0.2.12 (https://github.com/widdowquinn/pyani) revealed that NHP16-4001 exhibited the highest homology (97.5%) with *H. trogontum* ATCC700114^T^ (Fig. 3B). This value was greater than the cut-off value (95%) used for species identification (7). Thus, the blood culture-isolated NHP16-4001 strain was *H. trogontum*. The MICs were determined using the agar plate dilution method, as described previously (8). Briefly, the isolate was suspended in saline to achieve a turbidity equivalent to that of a McFarland 2.0 standard, and ~5 µL of inoculum was spotted onto Muller-Hinton agar (Becton, Dickinson, Franklin Lakes, NJ, USA) containing 5% horse blood and various concentrations of antimicrobial agents. The plates were incubated under microaerobic conditions with hydrogen obtained by the gas replacement method using an anaerobic gas mixture (H_2_, 10%; CO_2_, 10%; and N_2_, 80%) at 37°C for 2 days. The MICs of NHP16-4001 were high for ceftriaxone, clarithromycin, and ciprofloxacin (Table 1).

**TABLE 1:** MIC of *Helicobacter trogontum* and *H. cinaedi*

Antimicrobial agent	MIC (μg/mL)		MIC range (MIC90) for *H. cinaedi* (8)
	Present case	Mori et al. (9)	
Penicillin G		>1	
Amoxicillin	8	>1	
Amoxicillin/clavulanate			0.5–8 (8)
Ampicillin/sulbactam		8	
Piperacillin/tazobactam		≤16	
Ceftriaxone	32		
Ceftazidime		32	
Cefepime		16	
Cefoperazone/sulbactam		≤8	
Cefmetazole		>32	
Flomoxef		32	
Imipenem	0.063	≤0.25	0.031–0.125 (0.125)
Meropenem		≤0.25	
Gentamicin	1		0.125–1 (0.5)
Clindamycin		>4	
Clarithromycin	16		16 to >128 (>128)
Minocycline	0.25	≤0.25	0.016–0.25 (0.125)
Chloramphenicol		4	
Ciprofloxacin	4		16–128 (128)
Levofloxacin		2	

After discharge, she visited our hospital on day 15, as she continued to have a low-grade fever of 37.2°C–37.6°C and headache; however, the peripheral blood white blood cell count had improved to 9,550 /µL, neutrophil count was 69.0%, and C-reactive protein also improved to 0.03 mg/dL (Fig. 1). The bacteremia was considered cured. We confirmed the absence of recurrence upon her visit to our hospital 16 months post-discharge.

## DISCUSSION

We report a case of bacteremia in a 41-year-old woman working at a pig farm, in which the causative agent was *H. trogontum*. The infected organ could not be identified, and the patient was ultimately diagnosed with bacteremia.

WGS analysis suggested that the isolated strain NHP16-4001 was *H. trogontum*. The bacterium is a fusiform-to-slightly-spiral gram-negative cell with four to seven bipolar sheathed flagella (10). A PubMed search revealed that only two previous cases of human infection by *H. trogontum* have been reported: one in an immunocompetent patient (11) and the other in a patient with X-linked agammaglobulinemia (12). The first patient was a 31-year-old woman with a history of recurrent epigastralgia, vomiting, diarrhea, and weight loss over 8 years. The patient was diagnosed with acute colitis and bacteremia (11). The second patient was a 22-year-old man with symptoms of fever, malaise, and a painful skin lesion on the lower left extremity. The patient was diagnosed with bacteremia accompanied by skin lesions (12). Our case was similar to that of a 21-year-old man with sepsis who presented with abdominal pain, tenesmus, and fever (9). In the present case, the 16S rRNA gene sequence of NHP16-4001 showed 99.4% homology with that of the *Helicobacter* TMUC1514 strain, which was reported as the causative agent of bacteremia associated with enterocolitis in a young man (9). Although the genomic information is unavailable for the TMUC1514 strain, the previous case was possibly also caused by *H. trogontum*.

In *Helicobacter* species, the method for antimicrobial susceptibility testing is only established in *H. pylori*; thus, no standard method exists for the antimicrobial susceptibility testing of enterohepatic *Helicobacter* species. Therefore, we applied the antimicrobial susceptibility testing method for *H. cinaedi* reported in a previous study (8) to perform the susceptibility testing in this study. In our case, the MIC for ceftriaxone was high (Table 1). The TMUC1514 strain, which is probably *H. trogontum*, revealed high MICs of cephems, including cefmetazole (Table 1). In contrast, the infections caused by the TMUC1514 strain were treated with cefmetazole alone (Table 2) (9). Since meningoencephalitis was suspected in the case reported here, the patient was treated using ceftriaxone, and the patient’s condition improved. In the case of *H. cinaedi*, which is a prevalent enterohepatic *Helicobacter* species causing bacteremia in Japan, the MIC_90_ of ceftriaxone was ~32 µg/mL (Table 1) (8); despite this, several cases have been successfully treated with ceftriaxone. However, considering the lower MIC for amoxicillin compared to ceftriaxone, penicillin-based antibacterial drugs are suitable for enterohepatic *Helicobacter* species treatment, including this case.

**TABLE 2:** Clinical characteristics of patients with *Helicobacter trogontum* infection^*a*^

Case	Age (yr)	Sex	Occupation	Immunocompromised host	Initial symptoms	Laboratory findings	Focus of infection	Treatment and duration	Prognosis
Present case	41	Female	Pig farming	No	Headache, nausea, general fatigue, chills, fever	WBC: 15,630/µL; Neut: 14,270/µL; CRP: 0.25mg/dL	Bacteremia	CRO iv 7 d, VAN iv 5 d,ACV iv 4 d	Recovered
Dutasta et al. (11)	31	Female	No information	No	Abdominal pain, diarrhea, chills, fever	WBC: 13,200/µL; Neut: 11,400/µL; CRP: 19.1 mg/dL	Acute colitis, bacteremia	CIP, MTZ po 7 d	Recovered
Fjordside et al. (12)	22	Male	Pig farming	Yes, X-linked agammaglobulinemia	Painful skin lesion, malaise, fever	Neut: 6,040/µL; CRP: 13.2 mg/dL	Skin lesion, bacteremia	TZP iv 4 d, DOX po 10 d	Repeated recurrences
Mori et al. (9)	21	Male	No information	No	Abdominal pain, tenesmus, fever	WBC: 14,200/µL; CRP: 7.9 mg/dL	Enterocolitis, sepsis	CMZ iv within 5 d	Recovered

^*a*^ Abbreviations: ACV, acyclovir; CIP, ciprofloxacin; CMZ, cefmetazole; CRO, ceftriaxone; CRP, C-reactive protein; DOX, doxycycline; iv, intravenous; MTZ, metronidazole; Neut, neutrophil count; po, per os; TZP, piperacillin-tazobactam; VAN, vancomycin; WBC, white blood cell count.

Enterohepatic *Helicobacter* species can infect both humans and animals, and their probable transmission between humans and animals may serve as a reservoir for the transmission of pathogenic microorganisms to human contacts (13). Furthermore, *H. trogontum* was isolated from the feces and stomachs of pigs (4). Of the three patients with reported *H. trogontum* infection, one worked on a pig farm and was responsible for feeding pigs and mucking out their pens, similar to our case patient (12) (Table 2). We hypothesize that the development of bacteremia was related to contact with pig feces on the patient’s farm. *H. trogontum* infection may be considered a zoonosis; however, further case reports are warranted before a definitive conclusion can be reached.

## Data Availability

The genomic assembly of NHP16-4001 was deposited under accession no. BAAFHN010000001-BAAFHN010000237.
